# Supplementary material for: A missense mutation in zinc finger homeobox-3 (ZFHX3) impedes growth and alters metabolism and hypothalamic gene expression in mice
Source: FASEB J. Author manuscript; Available in PMC 2024 Feb 3. (PMC7615594; doi:10.1096/fj.202201829R)
Supplement: Supplementary Figures [file EMS193749-supplement-Supplementary_Figures.pdf]

Supplementary Material

A missense mutation in zinc finger homeobox-3 (ZFHX3) impedes growth and alters metabolism and hypothalamic gene expression in mice.

Patrick M. Nolan [1] *, Gareth Banks [1, 2], Nora Bourbia [1, 3], Ashleigh G. Wilcox [1], Liz Bentley [1], Lee Moir [1], Lee Kent [1], Rosie Hillier [1, 3], Dana Wilson [4], Perry Barrett [4], Rebecca Dumbell [1, 2] **

1: MRC Harwell Institute, Mammalian Genetics Unit and Mary Lyon Centre, Harwell Campus, Oxfordshire, UK

2: Nottingham Trent University, School of Science and Technology, Clifton Lane, Nottingham, UK

3: Current address: UK Health Security Agency, Centre for Radiation, Chemical and Environmental Hazards (UKHSA RCE), Harwell Campus, Oxfordshire, UK

4: The Rowett Institute, University of Aberdeen, Aberdeen, UK

* Co-corresponding author pmnolan10@gmail.com

**Co-corresponding author rebecca.dumbell@ntu.ac.uk

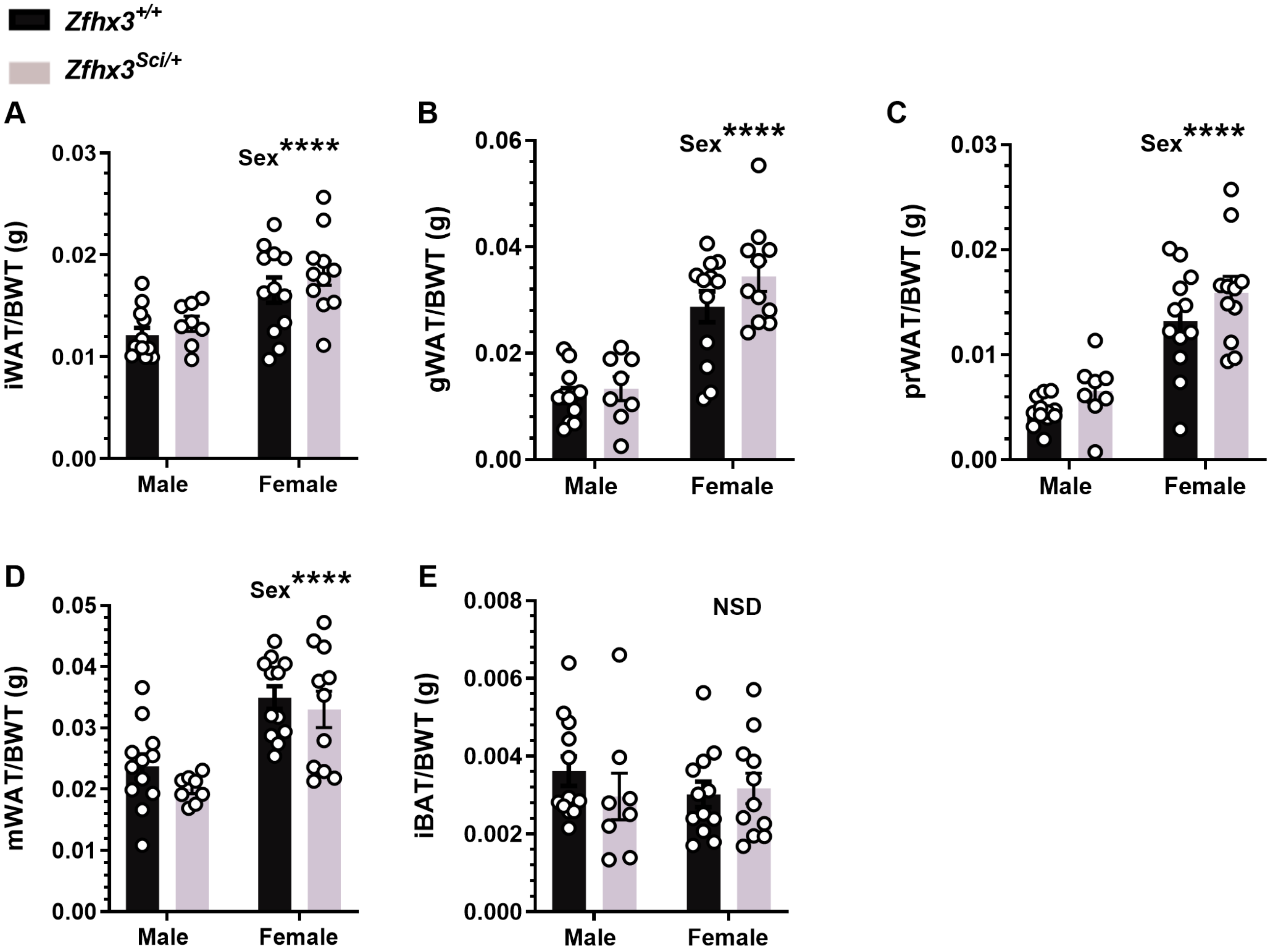

Supplemental Figure 1: One year old *Zfhx3*$^{Sci/+}$ mice fat pad mass do not differ to wildtype when corrected for body weight.

The *Sci* mutation in male and female mice does not alter fat pad mass / body weight (BWT). This corrected tissue mass is higher overall in female mice in al white adipose tissues measured (A-D), and sex did not affect brown adipose corrected mass (E). Plotted are mean ± SEM with individual values. Statistical comparison is by 2-way ANOVA, with overall comparisons indicated on the graphs. ****P<0.0001, NSD: no significant differences. iWAT: inguinal white adipose tissue, gWAT: gonadal white adipose tissue, prWAT: perirenal white adipose tissue, mWAT: mesenteric white adipose tissue, iBAT: interscapular brown adipose tissue.

Supplemental Table S1: Statistical Comparisons for Cumilative Food Intake Data (Figure 3). Stated are the significantly different (or approaching significance) ANOVA comparison statistics at each timepoint for the data presented in figure 3.

Figure	Comparison	F-Stat	P Value
3 A<br>Food Intake	Week 9 Sex<br>Week 9 Genotype	F (1, 19) = 9.909<br>F (1, 19) = 12.18	P=0.0053<br>P=0.0024
3 A<br>Food Intake	Week 10 Sex<br>Week 10 Genotype	F (1, 19) = 17.50<br>F (1, 19) = 14.85	P=0.0005<br>P=0.0011
3 A<br>Food Intake	Week 11 Sex<br>Week 11 Genotype	F (1, 19) = 19.42<br>F (1, 19) = 14.19	P=0.0003<br>P=0.0013
3 A<br>Food Intake	Week 12 Sex<br>Week 12 Genotype	F (1, 19) = 21.47<br>F (1, 19) = 12.64	P=0.0002<br>P=0.0021
3 A<br>Food Intake	Week 13 Sex<br>Week 13 Genotype	F (1, 19) = 21.47<br>F (1, 19) = 12.64	P=0.0002<br>P=0.0021
3 A<br>Food Intake	Week 14 Sex<br>Week 14 Genotype	F (1, 19) = 20.86<br>F (1, 19) = 11.06	P=0.0002<br>P=0.0035
3 A<br>Food Intake	Week 15 Sex<br>Week 15 Genotype	F (1, 19) = 21.02<br>F (1, 19) = 10.14	P=0.0002<br>P=0.0049
3 A<br>Food Intake	Week 16 Sex<br>Week 16 Genotype	F (1, 19) = 20.29<br>F (1, 19) = 10.09	P=0.0002<br>P=0.0050
3 A<br>Food Intake	Week 17 Sex<br>Week 17 Genotype	F (1, 19) = 18.35<br>F (1, 19) = 8.977	P=0.0004<br>P=0.0074
3 A<br>Food Intake	Week 18 Sex<br>Week 18 Genotype	F (1, 19) = 19.54<br>F (1, 19) = 10.14	P=0.0003<br>P=0.0049
3 A<br>Food Intake	Week 19 Sex<br>Week 19 Genotype	F (1, 19) = 19.70<br>F (1, 19) = 9.839	P=0.0003<br>P=0.0054
3 A<br>Food Intake	Week 20 Sex<br>Week 20 Genotype	F (1, 21) = 14.15<br>F (1, 21) = 11.97	P=0.0011<br>P=0.0023
3 B<br>Bodyweight	Week 8 Sex<br>Week 8 Genotype	F (1, 42) = 4.721<br>F (1, 42) = 2.144	P=0.0355<br>P=0.1506
3 B<br>Bodyweight	Week 9 Sex<br>Week 9 Genotype	F (1, 42) = 13.33<br>F (1, 42) = 4.701	P=0.0007<br>P=0.0359
3 B<br>Bodyweight	Week 10 Sex<br>Week 10 Genotype	F (1, 42) = 21.39<br>F (1, 42) = 4.813	P<0.0001<br>P=0.0338
3 B<br>Bodyweight	Week 11 Sex<br>Week 11 Genotype	F (1, 42) = 23.35<br>F (1, 42) = 5.084	P<0.0001<br>P=0.0294
3 B<br>Bodyweight	Week 12 Sex<br>Week 12 Genotype	F (1, 42) = 27.12<br>F (1, 42) = 4.652	P<0.0001<br>P=0.0368
3 B<br>Bodyweight	Week 13 Sex<br>Week 13 Genotype	F (1, 42) = 27.06<br>F (1, 42) = 4.782	P<0.0001<br>P=0.0344
3 B<br>Bodyweight	Week 14 Sex<br>Week 14 Genotype	F (1, 42) = 26.07<br>F (1, 42) = 3.509	P<0.0001<br>P=0.0680
3 B<br>Bodyweight	Week 15 Sex<br>Week 15 Genotype<br>Week 15 Interaction	F (1, 42) = 21.00<br>F (1, 42) = 3.307<br>F (1, 42) = 4.145	P<0.0001<br>P=0.0761<br>P=0.0481
3 B<br>Bodyweight	Week 16 Sex<br>Week 16 Genotype	F (1, 42) = 19.12<br>F (1, 42) = 3.660	P<0.0001<br>P=0.0626
3 B<br>Bodyweight	Week 17 Sex<br>Week 17 Genotype	F (1, 42) = 15.02<br>F (1, 42) = 3.310	P=0.0004<br>P=0.0760
3 B<br>Bodyweight	Week 18 Sex<br>Week 18 Genotype<br>Week 18 Interaction	F (1, 42) = 13.87<br>F (1, 42) = 3.903<br>F (1, 42) = 4.342	P=0.0006<br>P=0.0548<br>P=0.0433

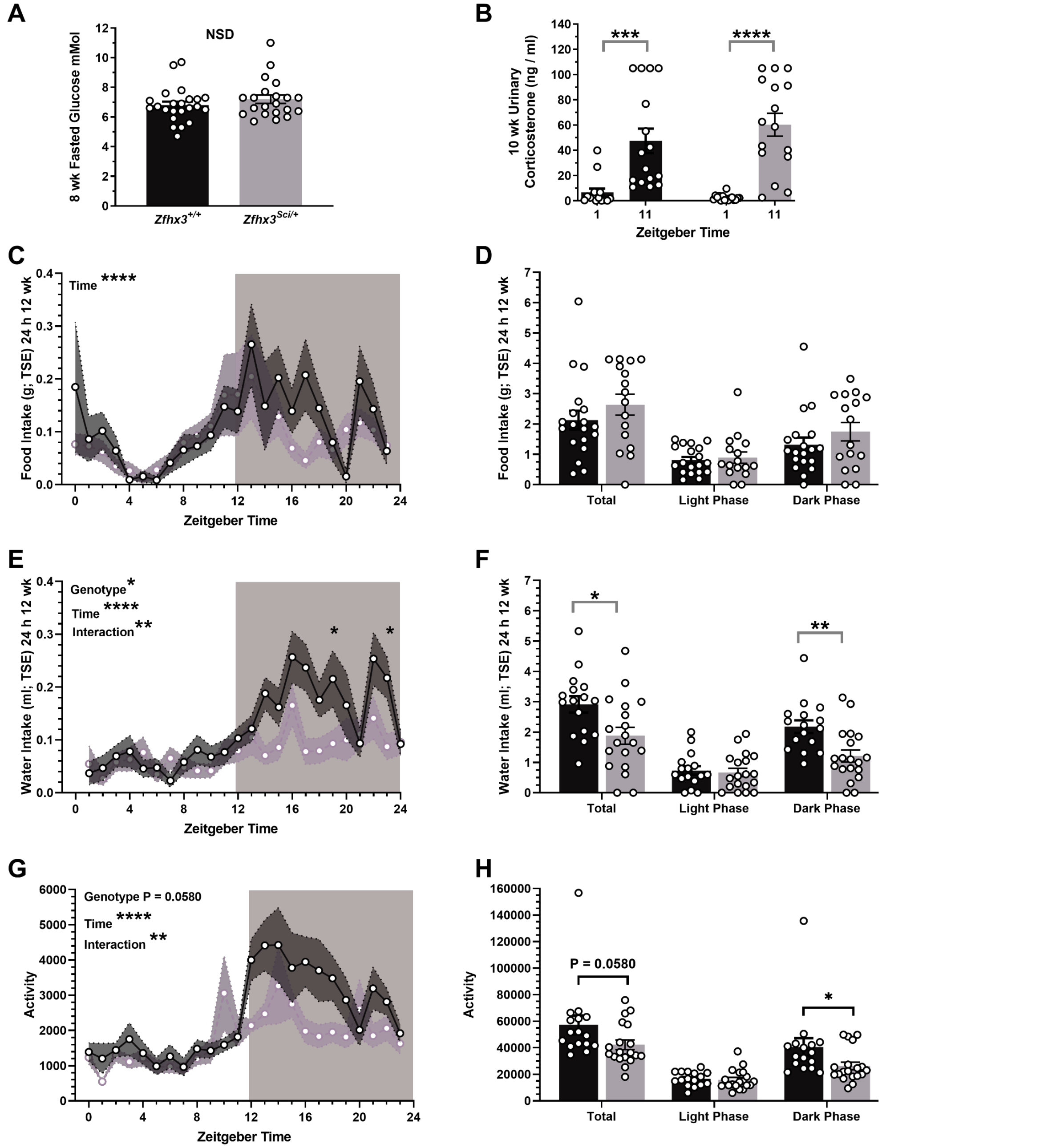

Supplemental Figure 2: 8 week blood glucose, 10 week urinary corticosterone and 24 h food and water intake data from TSE Phenomaster Calorimetry in 12 week old female *Zfhx3*$^{Sci/+}$ mice.

Fasted blood glucose was not altered in *Zfhx3*$^{Sci/+}$ mice at 8 weeks old (E), and neither was urinary corticosterone at 10 weeks, with peak and trough values maintained (F). Food intake in 1 h bins (C) total intake over 24 h, and light and dark phase only (D) was not altered by genotype during the sampling period in TSE Phenomaster metabolic cages, carried out in the second 24 h single housed in metabolic cages, at 12 weeks of age. However, water intake was significantly reduced overall in *Zfhx3*$^{Sci/+}$ mice, with an interaction between time and genotype (E) since water intake was significantly reduced in the dark phase and not the light phase (F). In the 24 h period, activity approached significant reduction in the *Zfhx3*$^{Sci/+}$ mice and there was a significant intereaction with time of sampling but this did not reach significance when considered in hourly bins (G), however when considering dark phase data only, this did the *Zfhx3*$^{Sci/+}$ mice did achieve significantly less activity (H). Plotted are mean ± SEM with (A, B, D, F, H) or without (C, E, G) individual values. Statistical comparison is by Mann-Whitney (A,B), Wilcoxon matched – pairs tests (between timepoints, B), or 2-way ANOVA, with overall comparisons indicated on the graphs (C, E, G), or unpaired t-test (D, F, H); ****P<0.0001, **P<0.01, *P<0.05.

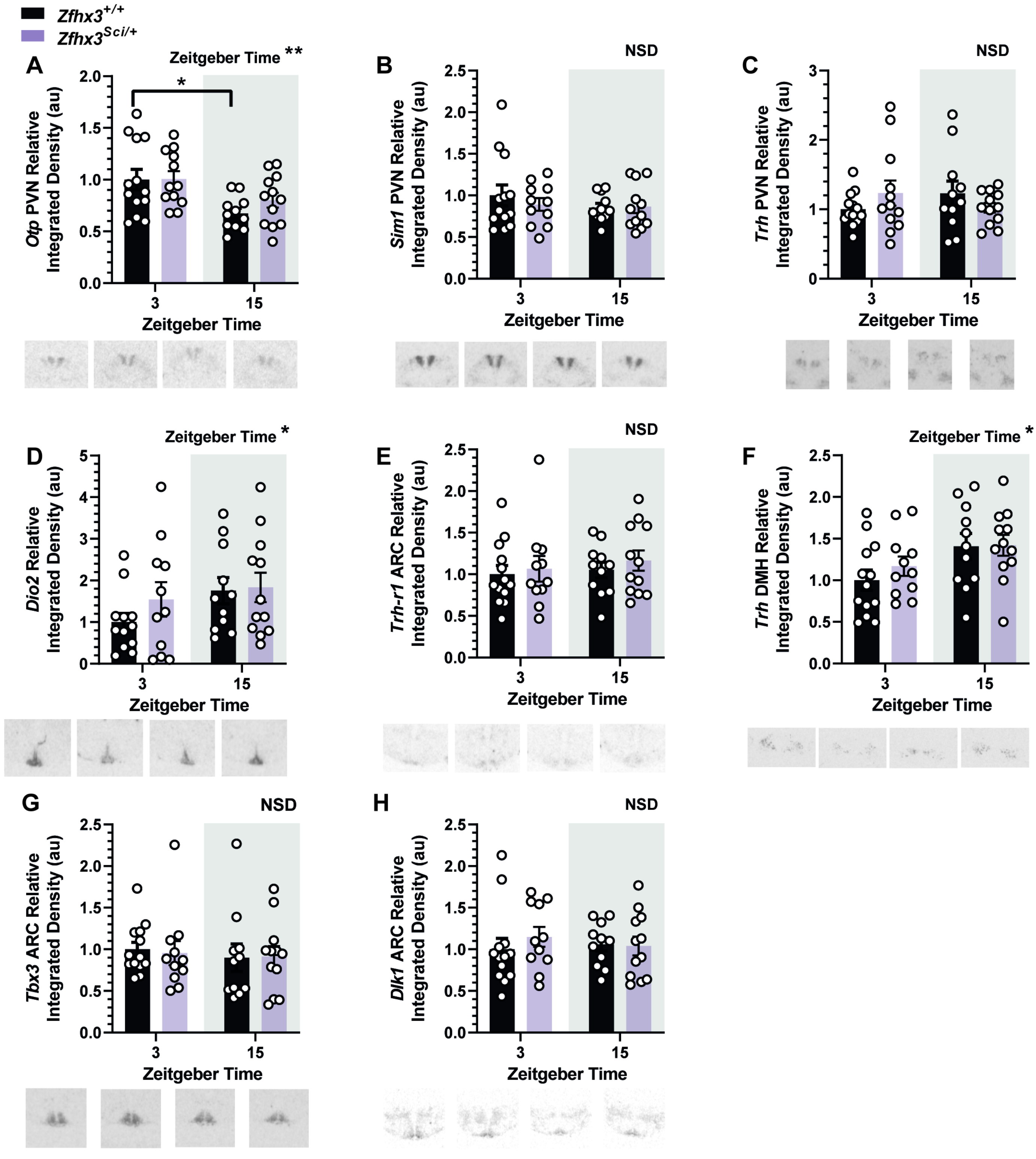

Supplemental figure 3: Hypothalamic candidate gene expression not altered in female *Zfhx3*$^{Sci/+}$ mice

Expression of *Otp* in the paraventricular nucleus (PVN) was reduced at ZT 15 (A), *Sim1* (B) and *Trh* (B) expression in the PVN were not altered by genotype or time. Expression of *Dio2* in the ventricular ependymal layer increased overall at ZT 15 (D) *Trh-r1* expression in the ARC was unaltered by time or genotype (E), while *Trh* expression in the dorsomedial hypothalamus was increased at ZT 15 (F), *Tbx3* (G) and *Dlk1* (H) expression in the ARC were unaltered by time or genotype. Plotted are mean ± SEM with individual values overlaid. Example images are shown beneath each plot. Comparisons are by 2-way ANOVA with Šídák's multiple comparison tests indicated where appropriate. *Dio2* data were log transformed for statistical comparison. * $P < 0.05$, ** $P < 0.01$, NSD: no significant differences. N = 10 – 12.
